# Associations between cardiovascular disease risk factors and spinal pain may be moderated by sex and health-related physical activity (CHAMPS Study-DK)

**DOI:** 10.1371/journal.pone.0277991

**Published:** 2022-11-21

**Authors:** Amber M. Beynon, Niels Wedderkopp, Charlotte Leboeuf-Yde, Jan Hartvigsen, Bruce F. Walker, Jeffrey J. Hébert

**Affiliations:** 1 Department of Chiropractic, Faculty of Medicine, Health and Human Sciences, Macquarie University, Sydney, New South Wales, Australia; 2 College of Science, Health, Engineering and Education, Murdoch University, Perth, Western Australia, Australia; 3 Research Unit of Paediatrics, Department of Clinical Research, Faculty of Health Sciences, University of Southern Denmark, Odense, Denmark; 4 Faculty of Kinesiology, University of New Brunswick, Fredericton, New Brunswick, Canada; 5 Department for Regional Health Research, University of Southern Denmark, Odense, Denmark; 6 Center for Muscle and Joint Health, Department of Sports Science and Clinical Biomechanics, University of Southern Denmark, Odense, Denmark; 7 Chiropractic Knowledge Hub, Odense, Denmark; University of Virginia, UNITED STATES

## Abstract

**Background:**

Spinal pain has been previously linked with cardiovascular disease risk factors in children. This study investigated the prospective associations between cardiovascular disease risk factors and non-traumatic spinal pain occurrences in children, and examined the moderating role of sex and health-related physical activity in these relationships.

**Methods:**

We used prospective data from the Childhood Health, Activity, and Motor Performance School Study Denmark (CHAMPS Study-DK). The exposure variables were a clustered cardiovascular risk score and homeostasis assessment model-estimated insulin resistance (HOMA-IR) score collected in 2008 and 2010. The spinal pain outcome comprised the number of weeks of non-traumatic spinal pain from 2008–2010 and 2010–2012. Potential confounders included age, sex, and time spent in moderate-to-vigorous intensity physical activity. We constructed age-adjusted mixed negative binominal regression models to investigate the prospective associations of cardiovascular disease risk factors and non-traumatic spinal pain, while considering the potential moderating roles of sex and physical activity in these relationships.

**Results:**

Girls with *low* HOMA-IR scores and boys with *low* clustered cardiovascular disease risk scores, who engaged in *higher* levels of moderate-to-vigorous physical activity, reported more weeks of spinal pain, compared to girls with *high* HOMA-IR scores (*p* = 0.001) and boys with *high* clustered cardiovascular disease risk scores (*p* = 0.024). whereas boys with *higher* clustered cardiovascular disease risk who had *less* time in moderate-to-vigorous physical activity reported more weeks of spinal pain than boys with *low* clustered cardiovascular disease risk score (*p* = 0.024).

**Conclusion:**

Our results show that cardiovascular disease risk factors are related to future occurrences of non-traumatic spinal pain. However, these relationships appear complex and dependent on the nature of the interactions with sex and physical activity.

## Introduction

Spinal pain is a significant public health problem. Even during adolescence, low back pain is ranked as one of the top ten causes of years lived with disability [1], and adolescents with low back pain have a higher risk of reporting low back pain in adulthood [2]. Musculoskeletal conditions commonly co-occur with other chronic diseases as part of multimorbidity [3, 4]. Multimorbidity is the co-existence of two or more diseases within an individual with the assumption that none of the diseases are more central, or take precedence, over the others [5, 6].

Cardiovascular disease is often one part of multimorbidity, and is the leading source of worldwide mortality [7]. We are unaware of other studies that have investigated the moderating role of health-related physical activity in the relationship between cardiovascular disease risk factors and spinal pain. However, several studies have evaluated these relationships separately. Cardiovascular disease is associated with low back pain in adults [8] and cardiovascular disease risk factors are more prevalent in people reporting high-intensity chronic pain [9]. Spinal pain is associated with cardiovascular disease risk factors in children [10] and a recent systematic review suggested a causal link between cardiovascular disease and musculoskeletal conditions including osteoarthritis (e.g., knee and hip) and back pain [3]. Considering other illnesses within comparable young populations, associations have been identified between early life chronic illnesses and back pain in young populations [11, 12]. A plausible biological link between these disorders may be that there is an inflammation-associated activation of the hypothalamic-pituitary-adrenal axis [13]. Dysregulation of this axis could lead to overactive responses to later psychosocial or mechanical stressors and overall hypersensitivity, resulting in pain [13]. This activation in the hypothalamic-pituitary-adrenal axis has been found to be associated with higher levels of cardiovascular disease risk factors [14]. The literature also demonstrations a higher prevalence of back pain with female sex [15, 16].

Clustering of cardiovascular disease risk factors begins in childhood and continues into adulthood [17–19]. Therefore, childhood is an opportune time to reduce modifiable cardiovascular disease risk factors not only to reduce risk of cardiovascular disease but, if a causal link exists, potentially also other conditions such as spinal pain.

This study aimed to 1) investigate the prospective associations between cardiovascular disease risk factors and non-traumatic spinal pain occurrences in children, and 2) examine the moderating roles of sex and health-related physical activity in these relationships. Non-traumatic pain is reported pain not arising from a traumatic aetiology (not fractures, sprain, contusion but rather “back pain” of unknown cause). We hypothesized that children with greater cardiovascular disease risk factors would be at increased risk of developing spinal pain and that sex and health-related physical activity would moderate this relationship.

## Methods

### Study design and ethics permissions

In this prospective cohort study, we used data from the participants of the Childhood Health, Activity, and Motor Performance School Study Demark (CHAMPS study-DK) [20]. All students from ten public primary schools were included on a rolling basis from October 2008. The sample comprised children from 6 to 11 years of age at the time of enrolment [21, 22]. We excluded children with serious chronic health conditions that precluded their participation in the study activities; three children were excluded based on this criterion: one child with dwarfism, one child with a congenital heart malformation, and one child with cerebral palsy.

The current study analyses were conducted in two phases (including the same participants in both phases) to account for the two exposure periods and subsequent spinal pain measurements. Phase one included cardiovascular disease risk factors sampled in October 2008 and spinal pain data collected weekly during November 2008 to November 2010. Phase two included cardiovascular disease risk factors sampled in October 2010 and spinal pain data collected weekly during November 2010 to November 2012.

Ethics approval was obtained from the Regional Scientific Committee of Southern Denmark for the CHAMPS study-DK (ID S20080047) and the study was registered with the Danish Data protection Agency, as stipulated by Danish law J.nr 2008-41-2240. Written informed consent was obtained from parents. Every child and parent also gave verbal consent prior to enrolment. Ethics approval for the current analyses was provided by Murdoch University Human Research Ethics Committee (Approval number: 2019/012). Data was obtained after applying for accessing through the CHAMPS Study Steering Committee. All data were fully anonymised before we accessed them for this current analyses.

### Cardiovascular disease risk factors

Fasting blood samples were obtained between 8.00 to 10.30 AM, stored on ice, and transported to the laboratory within four hours, where they were pipetted, centrifuged, and stored at -80 degrees Celsius [23]. Biochemical serum markers included: total cholesterol, high-density lipoprotein cholesterol (HDL-C), total cholesterol: HDL-C ratio, triglycerides, glucose, and insulin [23]. The homeostasis assessment model-estimated insulin resistance (HOMA-IR) score was calculated as insulin (μU/ ml) × glucose (mmol/l)/22.5 [23, 24].

Systolic blood pressure was measured with an automated blood pressure monitor [Welch Allyn® (New York, USA) vital signs monitor 300 series with FlexiPort™]. Blood pressure was taken seated after the participants had rested for five minutes and were recorded at 1-minute intervals until three stable measurements or five total measurements were obtained. The mean of the final three measurements was used for analysis [23].

The primary exposure variable was a clustered cardiovascular risk score, which has been reported as a better assessment of cardiovascular health in children compared to a single risk factor [25]. The clustered cardiovascular risk score was calculated by summing the standardized values of systolic blood pressure, total cholesterol: HDL-C ratio, log triglycerides, and log HOMA-IR [26, 27]. All scores were then converted to positive values, with larger scores representing higher levels of cardiovascular disease risk [23]. The second exposure variable was the log HOMA-IR score alone. We calculated tertiles for each exposure variable to distinguish between the children with lower- and higher-risk values.

### Spinal pain outcome

Spinal pain was defined as any pain in the neck, mid-back, and/or low back during the past week. Spinal pain data were collected through weekly text messages over approximately four years. When pain was reported, the parents were contacted by telephone the following day. If pain persisted at that time, the child was examined by a clinician [21]. ICD codes were used to classify the spinal pain diagnosis at the time of examination and occurrences were classified as traumatic or non-traumatic [28]. Research staff examined linked medical records for additional information. Additional details of the spinal pain measurement procedures have been reported previously [29].

In the current analyses, we excluded all occurrences of diagnosed spinal pain arising from a traumatic aetiology (e.g., fracture, sprain, contusion). Therefore, the spinal pain outcome comprised the total number of weeks of non-traumatic spinal pain (sometimes referred to as non-specific neck and back pain, soft tissue pain, facet syndrome) occurring in each of the two study phases. To be included in the analysis, participants needed to have at least 60% valid reporting of spinal pain data during the respective two-year phase. For example, to be included in phase one, participants needed at least 60% valid reporting of spinal pain data in phase one, irrespective of reporting in phase two.

### Covariates

Potential moderators and confounders included age, sex, and time spent in moderate-to-vigorous intensity physical activity (health-related physical activity). Demographic information was collected through a questionnaire at baseline. Physical activity was measured at baseline of each study period using Actigraph GTX3 accelerometers [20, 30]. Participants wore the accelerometer at the right hip, using a customised elastic belt, for seven consecutive days during waking hours (except when swimming or bathing). Data on physical activity were included if the participant accumulated at least ten hours of wear time on four or more days. We applied standard cut-points to identify moderate and vigorous physical activity intensities and isolated the proportion of the day in moderate-to-vigorous intensity physical activity (health-related physical activity) [20, 30]. These covariates were chosen due to their potential associations with back pain [15, 16] and cardiovascular disease risk factors [31].

### Statistical analysis

Demographic data were summarised with descriptive statistics. We log-transformed exposure variables with non-normal distributions.

In the first analysis (phase one), we used the baseline cardiovascular disease risk factors sampled in September–October 2008 as predictors for the number of weeks with non-traumatic spinal pain from November 2008 to November 2010. In the second analysis (phase two), we used the cardiovascular disease risk factors sampled in September–October 2010 as predictors for the number of weeks with non-traumatic spinal pain from November 2010 to November 2012.

To examine the prospective associations between childhood cardiovascular disease risk factors and spinal pain occurrences in each study phase (aim one), we constructed separate, mixed negative binominal regression models. Negative binomial models are well suited for zero-inflated count data (weeks with spinal pain) [32]. To account for the hierarchical nature of this school-based study, we included each child’s school class identifier as a random effect in all models. Age was included as a potential confounder and sex was added as a potential modifier by adding an interaction term with the cardiovascular disease risk factor. Model results were reported with unstandardized beta coefficients (β) and 95% confidence intervals (CI), stratified by sex. The lowest tertile group was used as the reference for the HOMA-IR and clustered cardiovascular disease risk factor exposure groups.

To investigate for additional moderating by health-related physical activity in the relationship between cardiovascular disease risk factors and spinal pain (aim two), we repeated the same modelling procedure and included a three-way interaction between the cardiovascular risk factor (HOMA-IR and clustered cardiovascular disease risk factors), sex, and time in moderate-to-vigorous physical activity. We examined the nature of these interactions by stratifying model results on sex, estimating the predicted margins, and plotting these results graphically.

Data were analysed using and Stata/SE version 15 software (StataCorp, TX). *P* values less than 0.05 were considered statistically significant.

## Results

Overall, 1630 participants participated in the study. In the first study phase, the study sample consisted of 1104 children (53% female) with a mean (SD) age of 8.4 (1.4) years and the second phase included 1291 children (52% female) with a mean (SD) age of 10.4 (1.4) years (Table 1).

**Table 1:** Baseline descriptive demographics and cardiovascular risk variables for each study phase.

Variable	2008 (phase 1)		2010 (phase 2)	
	(n)	Mean (SD)	(n)	Mean (SD)
**Age (yr.)**	Girls (572)	8.3 (1.4)	Girls (588)	10.3 (1.4)
	Boys (527)	8.4 (1.4)	Boys (541)	10.4 (1.4)
Body mass index (kg/m^2^)	Girls (571)	16.4 (2.1)	Girls (588)	17.5 (2.5)
	Boys (522)	16.3 (2.0)	Boys (540)	17.1 (2.3)
**Insulin (μU/mL)**	Girls (479)	3.9 (2.9)	Girls (447)	5.1 (2.9)
	Boys (447)	3.4 (2.1)	Boys (429)	5.0 (6.6)
**Glucose (mmol/L)**	Girls (478)	4.5 (0.4)	Girls (447)	4.7 (0.3)
	Boys (447)	4.7 (0.8)	Boys (429)	4.9 (0.8)
**HOMA-IR**	Girls (478)	0.8 (0.6)	Girls (447)	1.1 (0.7)
	Boys (447)	0.7 (0.6)	Boys (429)	1.3 (4.5)
**Systolic BP (mm Hg)**	Girls (555)	101.1 (8.1)	Girls (588)	102.0 (8.2)
	Boys (510)	101.5 (8.7)	Boys (541)	102.3 (8.1)
**Total Cholesterol (mg/dL)**	Girls (478)	174.5 (28.8)	Girls (447)	167.8 (26.2)
	Boys (447)	167 (25.7)	Boys (429)	163.2 (25.3)
**HDL Cholesterol (mg/dL)**	Girls (478)	63.0 (13.7)	Girls (447)	62.1 (12.8)
	Boys (447)	66.1 (13.3)	Boys (429)	64.3 (14.1)
**Total:HDL-C (mg/dL)**	Girls (478)	2.9 (0.7)	Girls (447)	2.8 (0.7)
	Boys (447)	2.6 (0.6)	Boys (429)	2.6 (0.6)
**LDL Cholesterol (mg/dL)**	Girls (478)	99.3 (26.7)	Girls (447)	93.5 (23.8)
	Boys (447)	90.6 (23.7)	Boys (428)	87.4 (22.4)
**Triglycerides (mg/dL)**	Girls (478)	60.8 (23.7)	Girls (447)	59.0 (26.6)
	Boys (447)	52.1 (20.9)	Boys (428)	54.4 (27.6)
**Clustered CV risk score**	Girls (467)	12.8 (2.6)	Girls (447)	16.1 (2.6)
	Boys (433)	11.8 (2.5)	Boys (428)	15.4 (2.9)
**MVPA (% of day)**	Girls (591)	7.4 (2.3)	Girls (608)	7.2 (2.6)
	Boys (519)	9.0 (2.5)	Boys (532)	9.6 (3.1)

HOMA-IR: homeostasis assessment model-estimated insulin resistance, BP: blood pressure, HDL-C: high-density lipoprotein cholesterol, LDL-Cholesterol: low-density lipoprotein cholesterol, CV: cardiovascular, MVPA: moderate-to-vigorous physical activity.

Sixty-three percent of children reported one or more occurrences of any type of spinal pain. The prevalence of non-traumatic spinal pain was very similar between phase one and two, however, there the mean duration of weeks with spinal pain was 2 weeks longer in phase two with a statistically significant difference in girls (*p* = 0.0002) but not boys, and more children reported four or more weeks with pain (Table 2).

**Table 2:** Number of weeks of reported spinal pain and non-traumatic spinal pain for each study phase.

	**Phase 1 (Nov 2008-Nov 2010)**			
	(n)	Mean (SD)	Median (IQR)	Range
**Weeks with spinal pain**	Girls (587)	1.9 (5.7)	0 (1)	0 to 62
	Boys (517)	1.5 (6.0)	0 (1)	0 to 75
**Weeks with non-traumatic spinal pain**	Girls (587)	1.7 (5.4)	0 (1)	0 to 62
	Boys (517)	1.3 (5.9)	0 (1)	0 to 75
	**Phase 2 (Nov 2010-Nov 2012)**			
	(n)	Mean (SD)	Median (IQR)	Range
**Weeks with spinal pain**	Girls (676)	3.3 (8.5)	0 (2)	0 to 91
	Boys (615)	2.0 (6.7)	0 (2)	0 to 90
**Weeks with non-traumatic spinal pain**	Girls (676)	3.1 (8.5)	0 (2)	0 to 91
	Boys (615)	1.9 (6.8)	0 (2)	0 to 90

### Cardiovascular disease risk factors and future spinal pain

In phase one, there were significant two-way interactions between log-HOMA-IR and sex (*p* = 0.029), and between clustered cardiovascular disease risk and sex (*p* = 0.006). We found that girls with moderate log HOMA-IR (tertile two) were less likely to experience non-traumatic spinal pain compared to girls with low log HOMA-IR (tertile one) (β [95% CI] = -0.83 [-1.57, -0.08]) (Table 3).

**Table 3:** Associations between HOMA-IR and Clustered cardiovascular disease risk score and spinal pain.

**Cardiovascular disease risk factors 2008**	**N**	Weeks with spinal pain Nov 2008-Nov 2010 Tertile 2 *beta coefficients (95% CI)	Weeks with spinal pain Nov 2008-Nov 2010 Tertile 3 *beta coefficients (95% CI)
**Log HOMA-IR**	Girls = 440	**-0.83 (-1.57, -0.08)**	-0.80 (-1.65, 0.05)
	Boys = 406	0.04 (-0.74, 0.83)	0.25 (-0.68, 1.17)
**Clustered CV risk score**	Girls = 433	-0.62 (-1.29, 0.05)	-0.00 (-0.83, 0.83)
	Boys = 397	0.30 (-0.29, 0.89)	0.60 (-0.36, 1.57)
**Cardiovascular disease risk factors 2010**	**N**	**Weeks with spinal pain Nov 2010-Nov 2012 Tertile 2** *beta coefficients (95% CI)	**Weeks with spinal pain Nov 2010-Nov 2012 Tertile 3** *beta coefficients (95% CI)
**Log HOMA-IR**	Girls = 370	-0.38 (-1.21, 0.45)	**-1.57 (-2.63, -0.51)**
	Boys = 367	0.42 (-0.37, 1.21)	0.28 (-0.87, 1.43)
**Clustered CV risk score**	Girls = 370	0.39 (-1.08, 1.86)	-0.47 (-2.02, 1.07)
	Boys = 366	-0.11 (-1.13, 0.91)	0.22 (-1.06, 1.50)

Tertile 1: reference group

HOMA-IR: homeostasis assessment model-estimated insulin resistance, CV: cardiovascular

*All models adjusted for age

Bolded results indicate statistically significant results.

In phase two, there was a significant two-way interaction between log-HOMA-IR and sex (*p* = 0.002) but not between clustered cardiovascular risk and sex (*p* = 0.465). We found girls with high log HOMA-IR (tertile three) were less likely to experience non-traumatic spinal pain than girls with low log HOMA-IR (tertile one) (β [CI] = -1.57 [-2.63, -0.51]). There were no other associations between the cardiovascular disease risk factors and spinal pain (Table 3).

### Cardiovascular disease risk factors, future spinal pain, and the moderating role of physical activity

**Fig 1:**
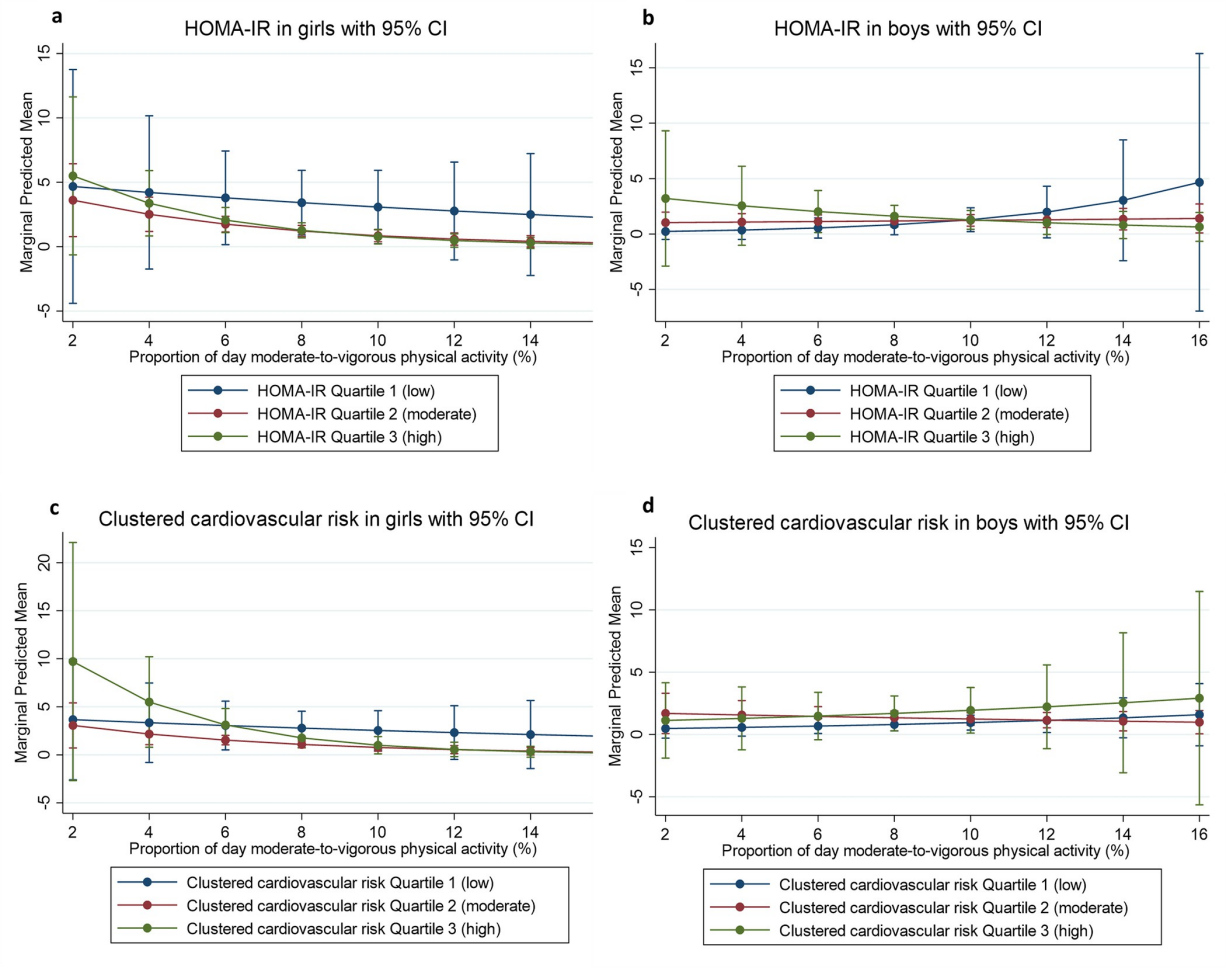
Predictive margins of HOMA-IR and clustered cardiovascular risk for phase 1. a. HOMA-IR in girls with 95% CI. b. HOMA-IR in boys with 95% CI. c. Clustered cardiovascular disease risk in girls with 95% CI. d. Clustered cardiovascular disease risk in boys with 95% CI. No significant 2-way interaction with cardiovascular disease risk factor and moderate-to-vigorous physical activity.

In phase one, there were no associations between the cardiovascular disease risk factors and non-traumatic spinal pain when accounting for the moderating role of health-related physical activity (Fig 1).

**Fig 2:**
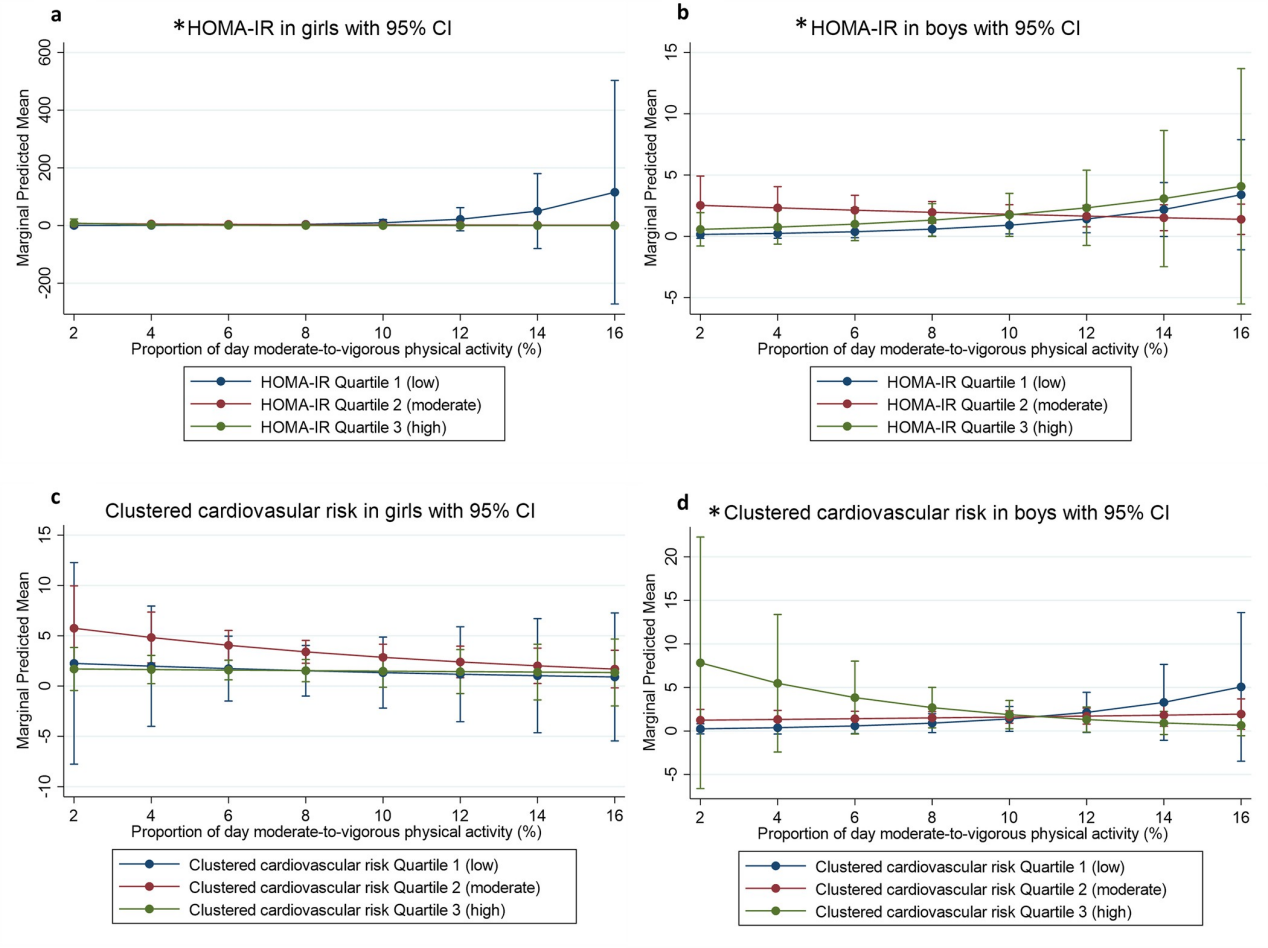
Predictive margins of HOMA-IR and clustered cardiovascular risk for phase 2. a. HOMA-IR in girls with 95% CI. b. HOMA-IR in boys with 95% CI. c. Clustered cardiovascular disease risk in girls with 95% CI. d. Clustered cardiovascular disease risk in boys with 95% CI. *Significant 2-way interaction with cardiovascular disease risk factor and moderate-to-vigorous physical activity.

In phase two, there was a significant three-way interaction (*p* = 0.009) between log-HOMA-IR, moderate-to-vigorous physical activity, and sex. Overall, girls with low HOMA-IR scores, who engaged in higher levels of moderate-to-vigorous physical activity, reported more weeks of spinal pain. Boys with lower clustered cardiovascular disease risk and more time in moderate-to-vigorous physical activity, reported more weeks with spinal pain. Further, boys with higher clustered cardiovascular risk, who had less time in moderate -to-vigorous physical activity also reported more weeks of spinal pain (Fig 2).

Specifically, in phase two, girls with low (tertile one) log HOMA-IR risk scores and more moderate-to-vigorous physical activity had a higher likelihood of non-traumatic spinal pain than girls with moderate (tertile two) and high (tertile three) log HOMA-IR scores (two-way interaction *p* = 0.001 to 0.009) (Fig 2A). Boys with low (tertile one) log HOMA-IR scores and more time in moderate-to-vigorous physical activity had a higher likelihood of non-traumatic spinal pain than boys with moderate (tertile two) log HOMA-IR scores (two-way interaction *p* = 0.033) (Fig 2B).

In phase two, there were no association found between clustered cardiovascular disease risk score and non-traumatic spinal pain in girls (Fig 2C). Boys with low (tertile one) clustered cardiovascular disease risk scores and more time in moderate-to-vigorous physical activity reported more weeks with non-traumatic spinal pain than boys with high (tertile three) cardiovascular disease risk scores (significant two-way interaction *p* = 0.024) (Fig 2D). Furthermore, boys with a high (tertile three) clustered cardiovascular disease risk score and less moderate-to-vigorous physical activity were more likely to experience non-traumatic spinal pain than boys with low (tertile one) clustered cardiovascular disease risk score (two-way interaction *p* = 0.024) (Fig 2D).

## Discussion

Boys and girls seem to react somewhat differently to moderate or vigorous physical activities depending on their cardiovascular risk profile, as a *low* cardiovascular risk profile (low clustered cardiovascular risk score and low HOMA-IR or) indicated a risk for spinal pain for both girls and boys who had higher levels of moderate-to-vigorous physical activity. For those with a *higher* cardiovascular risk profile, boys, but not girls, would report spinal pain also with less moderate or vigorous physical activity. However, these findings were observed only when the children were older. Throughout the literature there has been an increased reported incidence and prevalence of back pain with female sex and advancing age towards adolescence and young adulthood [15, 16], potentially there is a physiological process that occurs during puberty that may affect spinal pain.

A previous study reported that girls with spinal pain had greater clustered cardiovascular risk compared to girls without spinal pain, independent of health-related physical activity [10]. However, in that study, there was a lack of association between spinal pain and clustered cardiovascular risk in boys [10]. There is inconsistent evidence regarding the nature of the relationship between spinal pain and physical activity [15, 16, 33]. It has been previously suggested that the association between physical activity and low back pain should be considered on a continuum, a ‘U-shape distribution’ [34]. This has been supported by results from a population-based study which found cross-sectional associations between extremes of physical activity (high levels/intensity or low levels) with chronic low back pain, particularly in women [35]. Conversely, Heuch et al. [36] reported no evidence of a U-shaped relationship between physical activity and low back pain. There was a call for longitudinal studies to consider this relationship between low back pain and physical activity [37], and in our longitudinal analyses we found that physical activity moderates the association of cardiovascular disease risk factors and spinal pain in older children. Our analyses support the U-shape hypothesis and may explain the conflicting findings previously. A previous study found that children who participated in organised leisure-time sports had a decreased clustered cardiovascular disease risk compared to children who did not participate in organised leisure-time sports [23], further vigorous intensity physical activity has been found to be associated with increased spinal pain [28]. Perhaps children with low cardiovascular disease risk factors who engage in high amounts of physical activity report more spinal pain due to overuse or sporting injuries that have not been reported as traumatic. On the other hand, in children with higher levels of cardiovascular disease risk factors, there could be an inflammatory-associated activation of the hypothalamic-pituitary-adrenal axis [13, 14], which could lead to overactive responses to later psychosocial or mechanical stressors and overall hypersensitivity, resulting in pain [13]. Overall, our findings support the hypothesis that depending on an individual’s cardiovascular disease risk score, age and sex, too much or too little activity may be associated with increased spinal pain.

Strengths of the current study include its longitudinal design and large, representative cohort of children. We used uniquely robust measurements of spinal pain, cardiovascular disease risk factors, and physical activity. Spinal pain data were intensively collected with weekly text messaging, which likely reduces recall bias and resulted in high levels of participant engagement, with the average weekly response rate of 96.5% [21]. Although models were adjusted for potential modification and confounding, residual confounding is possible.

However, there are limitations to our study. Our study was exploratory and hypothesis generating. These results require confirmation before considering clinical or policy-related implications. In order to identify a causal relationship, at a minimum, the risk factor should be established before the onset of the disorder (spinal pain) [15]. As this was not an inception cohort, we cannot be certain there was an absence of spinal pain at baseline. Thus, we cannot make confident judgements about causation, as temporality has not been established. However, as spinal pain is uncommon in very young children [21], we expect that relatively few children had established spinal pain prior to enrolment in the study, lessening the risk of contamination of the outcome variable. Additional evidence is needed to judge the likely causal nature of these relationships between cardiovascular disease risk factors, physical activity, and spinal pain. We excluded children with serious chronic health conditions that precluded their participation in the study activities, this is based on the ability to participate in the study activities. We do not know if the children had any other medical conditions present that could have impacted the spinal pain measurement. Within this current analysis we do not know the amount or type of sport played by the children, we have earlier assessed the effect of physical activity and sport on back pain in children and found that sport participation does increase the risk of risk of traumatic back pain, but the incidence due to sport participation was overall still low, the only sports that had a relatively high incidence rate were horseback riding (2.45 per 1000 units of physical activity, 1 unit = 30 minutes) and tumbling gymnastics (1.25 per 1000 units of physical activity) [38]. Although we excluded traumatic spinal pain, participation in sport could impact spinal pain if they were dealing with a sports related injury. On the other hand, being physically active could potentially be of benefit. Gaining a better understanding on the types of physical activity or if participants engaged in sports and what types of sports may potentially assist in our understanding of why children with low cardiovascular disease risk factors who engage in more physical activity report more spinal pain. Future studies should also consider the types of physical activity and engagement in sports to help gain a better understanding of this potential relationship.

## Conclusion

Girls with low insulin resistance and boys with low clustered cardiovascular risk scores at baseline and who spent high amounts of time doing moderate-to-vigorous physical activity reported more weeks with spinal pain during the two year follow up. Boys with higher clustered cardiovascular risk with low time in moderate-to-vigorous physical activity also reported more weeks with spinal pain. Thus, there appears to be a relationship between insulin resistance, clustered cardiovascular risk factors and future spinal pain in children aged approximately 10 to 12 years of age and this relationship may be moderated by sex and health-related physical activity.
